# An aerosol challenge model of tuberculosis in Mauritian cynomolgus macaques

**DOI:** 10.1371/journal.pone.0171906

**Published:** 2017-03-08

**Authors:** S. A. Sharpe, A. D. White, L. Sibley, F. Gleeson, G. A. Hall, R. J. Basaraba, A. McIntyre, S. O. Clark, K. Gooch, P. D. Marsh, A. Williams, M. J. Dennis

**Affiliations:** 1 Public Health England, National Infection Service, Porton Down, Salisbury, SP4 0JG, United Kingdom; 2 The Churchill Hospital, Headington, Oxford, United Kingdom; 3 Department of Microbiology Immunology and Pathology, College of Veterinary Medicine and Biomedical Sciences, Colorado State University, Fort Collins, Colorado, United States of America; University of Pittsburgh, UNITED STATES

## Abstract

**Background:**

New interventions for tuberculosis are urgently needed. Non-human primate (NHP) models provide the most relevant pre-clinical models of human disease and play a critical role in vaccine development. Models utilising Asian cynomolgus macaque populations are well established but the restricted genetic diversity of the Mauritian cynomolgus macaques may be of added value.

**Methods:**

Mauritian cynomolgus macaques were exposed to a range of doses of *M*. *tuberculosis* delivered by aerosol, and the outcome was assessed using clinical, imaging and pathology-based measures.

**Results:**

All macaques developed characteristic clinical signs and disease features of tuberculosis (TB). Disease burden and the ability to control disease were dependent on exposure dose. Mauritian cynomolgus macaques showed less variation in pulmonary disease burden and total gross pathology scores within exposure dose groups than either Indian rhesus macaques or Chinese cynomolgus macaques

**Conclusions:**

The genetic homogeneity of Mauritian cynomolgus macaques makes them a potentially useful model of human tuberculosis.

## 1. Introduction

*Mycobacterium tuberculosis (M*. *tuberculosis)*, the causative agent of tuberculosis (TB), is responsible for 9 million new infections and 1.5 million deaths each year [1]. Tuberculosis is an enormous global health issue due to the rise in drug-resistant strains, the impact of co-infection with HIV, and the estimate that a third of the world’s population could be latently infected. Vaccination is widely accepted to be the most effective method for infectious disease control, and although the only licenced vaccine against *M*. *tuberculosis*, Bacille Calmette-Guréin (BCG), protects children from developing severe TB disease [2], the protection afforded to adults is limited [3], and it is unsuitable for use in people whose immune system is compromised, so more effective vaccines against *M*. *tuberculosis* are desperately needed.

Preclinical animal models are critical to the development of new vaccines, as studies of infectious challenge can be used to predict the effectiveness of vaccines in humans and they provide the opportunity to identify and validate correlates of protection. Due to their close similarity to humans, non-human primates offer the most relevant models of human diseases, and models of *M*. *tuberculosis* have been established in both rhesus macaque (*Macaca mulatta*) and cynomolgus macaque (*Macaca fascicularis*) species [4–6] with cynomolgus macaques showing an improved ability to control *M*. *tuberculosis*-induced disease [7, 8]. To date, the cynomolgus macaque models reported have employed Asian cynomolgus macaques from either China or the Philippines [9–12] and studies have shown these populations to exhibit a full spectrum of outcomes to *M*. *tuberculosis* challenge, from latent infection to the development of active progressive disease [4], which is likely to be a reflection of their wide genetic diversity.

Cynomolgus macaques are indigenous to mainland Southeast Asia, and surrounding islands such as Sumatra and the Philippines, but have also been introduced as an alien species in several locations, including the island of Mauritius. The Mauritian cynomolgus macaque population that descended from the small founder group consequently show a reduced genetic variability [13]. Consequently, differences in genetic composition [14] and phenotypic characteristics, such as body weight [15], sexual maturation rate [16], blood chemistry and haematology [17], and microbiome [18] compared with populations from within the species natural range of habitats in Asia have been reported. Furthermore, developments in the immunogenetic characterisation of Mauritian cynomolgus macaques have identified their value in the study of the role of host MHC genetics in immunogenicity and vaccine studies [19–27]. Thus, the Mauritian cynomolgus macaque has the potential to provide an important addition to the portfolio of macaque models of *M*. *tuberculosis*. A less genetically diverse population may, respond more reproducibly to infection, enhancing the ability to discriminate protective effects afforded by new vaccine candidates and facilitate the identification of much needed immune correlates of protection. The pilot study reported here aimed to establish an aerosol challenge model of TB in Mauritian cynomolgus macaques, and evaluate the effect of different challenge doses of *M*. *tuberculosis* on the clinical outcome.

## 2. Materials and methods

### 2.1 Experimental animals

Nine captive-bred cynomolgus macaques of Mauritian genotype aged between 2 and 4 years were obtained from an established UK breeding colony [28]. Absence of previous exposure to mycobacterial antigens (*M*. *tuberculosis* infection or environmental mycobacteria) was confirmed by the tuberculin test whilst the animals were still in their original breeding colony, and just prior to study start, by the IFN-γ based Primagam™ test kit (Biocor Animal Health Inc, Ohama, Nebraska, USA) and an *ex-vivo* IFN-γ ELISPOT (Mabtech AB, Nacka Strand, Sweden) to measure responses to mycobacterial antigens: PPD (Statens Serum Institute, Copenhagen, Denmark), and pooled 15-mer peptides of ESAT6 and CFP10 (Peptide Protein Research LTD, Fareham, UK). Animals were housed in compatible social groups, in accordance with the Home Office (UK) Code of Practice for the Housing and Care of Animals Used in Scientific Procedures (1989), and the National Committee for Refinement, Reduction and Replacement (NC3Rs) Guidelines on Primate Accommodation, Care and Use, August 2006 (NC3Rs, 2006) in cages approximately 2.5M high by 4M long by 2M deep. These cages were constructed with high level observation balconies and with a floor of deep litter to allow foraging. Further enrichment was afforded by the provision of toys, swings, feeding puzzles and DVDs for visual stimulation. In addition to standard old world primate pellets further food was provided by a selection of vegetables and fruit. Animals were sedated by intramuscular injection of ketamine hydrochloride (Ketaset, 100mg/ml, Fort Dodge Animal Health Ltd, Southampton, UK; 10mg/kg) for procedures requiring their removal from their housing. None of the animals had been used previously for experimental procedures and each socially compatible group was randomly assigned to a particular study treatment. All animal procedures and study design were approved by the Public Health England, Porton Down Ethical Review Committee, and authorized under an appropriate UK Home Office project license.

### 2.2 *M*. *tuberculosis* strain

The *M*. *tuberculosis* Erdman strain K01 used for challenge was provided by the CBER/FDA repository and prepared from frozen suspensions at a stated titre of 3.5 ± 1.5x10^8^ CFU/ml. For challenge, sufficient vials were thawed and diluted appropriately, as stated below, in sterile distilled water.

### 2.3 Aerosol exposure

Macaques were challenged by exposure to aerosols of *M*. *tuberculosis* as previously described [29–31]. Mono-dispersed bacteria in particles were generated using a 3-jet Collison nebuliser (BGI) and, in conjunction with a modified Henderson apparatus [32], delivered to the nares of each sedated primate via a modified veterinary anaesthetic mask. Challenge was performed on sedated animals placed within a ‘head-out’, plethysmography chamber (Buxco, Wilmington, North Carolina, USA) to enable the aerosol to be delivered simultaneously with the measurement of respired volume. The aerosol delivery process was designed to result in the deposition of a defined spread of target doses ranging from 22–250 CFU retained in the lungs (Table 1). This range of low and very high challenge doses was selected because the susceptibility of this species of macaque to TB was unknown. The number of bacilli deposited and retained in the lungs of macaques cannot be measured directly, and so quantification of the dose is calculated from the concentration of viable organisms in the aerosol (C_aero_) and the volume of aerosol inhaled by the animal. This ‘presented dose’ (PD) is the number of organisms that the animals inhale. C_aero_ is either measured directly using air sampling within the system or may be calculated using the concentration of organisms in the nebulizer (C_neb_) and a ‘spray factor’ that is a constant derived from data generated for the specific organism with identical aerosol exposure parameters. The calculations to derive the PD and the retained dose (the number of organisms assumed to be retained in the lung) have been described previously for high/medium aerosol doses [30, 31]. The assumed retained dose was calculated from the PD by applying a retention factor. Retention factors for rhesus macaques are described by Harper and Moreton [33].

**Table 1:** Aerosol challenge doses of *M*. *tuberculosis* delivered to Mauritian cynomolgus macaques.

Animal Identification number	Presented Dose range (CFU)	Presented Dose (CFU)	Estimated Retained Dose [30,31]. (CFU)
M982B	Very high [>1400]	1650	236
I131B		1624	232
M651		1544	221
M284D	High	522	75
	[500–1400]		
I319DB	Medium [250–500]	448	64
I347G		335	48
M054E		302	43
M064D	Low	242	35
M988E	[<250]	153	22

### 2.4 Clinical assessment

Animals were monitored daily for behavioural abnormalities including depression, withdrawal from the group, aggression, and changes in feeding patterns, respiration rate and the occurrence of cough. Animals were weighed, rectal temperature measured and examined for gross abnormalities on each occasion that required blood sample collection, aerosol challenge or euthanasia. Red blood cell (RBC) haemoglobin levels were measured using a HaemaCue haemoglobinometer (Haemacue Ltd, Dronfield, UK) to identify the presence of anaemia, and erythrocyte sedimentation rates (ESR) were measured using the Sediplast system (Guest Medical, Edenbridge, UK) to detect and monitor inflammation induced by infection with *M*. *tuberculosis*.

The time of necropsy, if prior to the end of the planned study period, was determined by experienced primatology staff and based on a combination of the following adverse indicators: depression or withdrawn behaviour, abnormal respiration (dyspnoea), loss of 20% of peak post-challenge weight, ESR levels elevated above normal (>20 mm), haemoglobin level below normal limits (< 100g/dL), increased temperature (>41^°^C) and abnormal thoracic radiograph. The range of adverse indicators used allowed application of humane intervention when individuals had progressed to moderate disease.

### 2.5 Immune response analysis

#### 2.5.1 Interferon-gamma (IFN-γ) ELISpot

The *M*. *tuberculosis*-specific immune response was evaluated at two weekly intervals throughout the study. Peripheral blood mononuclear cells were isolated from heparin anti-coagulated blood using standard methods. An IFN-γ ELISpot assay was used to estimate the numbers and IFN-γ production capacity of mycobacteria-specific T cells in PBMCs using a human/simian IFN-γ kit (Mabtech AB, Nacka Strand, Sweden), as described previously [30]. Cells were stimulated with PPD (10 μg/ml, SSI, Copenhagen, Denmark) or pools of overlapping 15mer peptides spanning CFP10, or ESAT6 (Peptide Protein Research Ltd, Wickham, UK).

#### 2.5.2 Quantification of secreted IFN-γ

IFN-γ production was measured using a whole blood ELISA as previously described [30]. In brief, heparinised blood was diluted 1 in 10 with serum-free medium (RPMI supplemented with L-glutamine, penicillin and streptomycin) and cultured with purified protein derivative from *M*. *tuberculosis* (PPD; 5 μg/ml, SSI, Copenhagen, Denmark), or mitogen Phytohaemaglutinin (PHA; Sigma-Aldrich, Dorset, UK) (5μg/l), or in medium alone for 6 days. Supernatants were harvested at day 6 and stored at -80^°^C. The quantity of IFN-γ in the supernatants was estimated using a commercially available human/monkey IFN-γ ELISA kit (Mabtech AB, Nacka Strand, Sweden). A purified human IFN-γ was used for the standard curve on each plate. The ELISA was developed using streptavidin and 3,3’,5,5’-tetramethylbenzidine (TMB) liquid substrate system (Sigma-Aldrich, Dorset, UK) and the reaction stopped with 2M sulphuric acid (May & Baker Ltd, Dagenham, UK). ELISA plates were read at 450nm. A standard curve was plotted for each plate and used to calculate the concentrations of IFN-γ in each sample.

### 2.6 Necropsy

Prior to euthanasia by intra-cardiac injection of a lethal dose of anaesthetic (Dolelethal, Vétoquinol UK Ltd, 140mg/kg), animals were anaesthetised and clinical data collected. Blood samples were taken. A post-mortem examination was performed immediately and gross pathological changes were scored using an established system based on the number and extent of lesions present in the lungs, spleen, liver, kidney and lymph nodes, as described previously [30, 31]. Samples of spleen, liver, kidneys and tracheobronchial, inguinal and axillary lymph nodes were removed and sampled for quantitative bacteriology. The lungs, including the heart and lung-associated lymph nodes, were removed intact and the lymph nodes examined for lesions. The complete lung set was fixed by intra-tracheal infusion with 10% neutral buffered formalin (NBF) using a syringe and 13CH Nelaton catheter (J.A.K. Marketing, York, UK). The catheter tip was inserted into each bronchus in turn via the trachea; the lungs were infused until they were expanded to a size considered to be normal inspiratory dimensions, and the trachea ligated to retain the fluid. The infused lung was immersed in 10% NBF. In addition, samples of kidneys, liver, spleen, and sub-clavicular, hepatic inguinal and axillary lymph nodes were fixed in 10% NBF.

### 2.7 Lung imaging

Thoracic radiographs (SP VET 3.2, Xograph Ltd) were acquired using mammography film (Xograph Imaging Systems Ltd, Tetbury, UK) before and every 2 weeks after exposure to *M*. *tuberculosis*. Evaluation of disease was performed by an experienced consultant thoracic radiologist blinded to the exposure dose and clinical status using a pre-determined scoring system based on the amount and distribution of infiltrate as previously described [31].

### 2.8 Magnetic resonance (MR) imaging

The *ex-vivo* expanded, fixed lungs were set in 2% agarose (Sigma-Aldrich, UK) and images were collected using a 3.0 T 750 MR Scanner (General Electric Healthcare, Milwaukee, WI, USA) as described previously [31]. This enabled evaluation of the pulmonary disease burden at the end of the study period. Lung lesions were identified in MR images from their signal intensity and nodular morphology relative to normal lung parenchyma.

### 2.9 Lesion analysis / quantification (stereology)

Lung lesions were identified on MR images based on their signal intensity and nodular morphology relative to more normal lung parenchyma. The total lung and lesion volume relative to the fixed tissue was determined using the Cavalieri method applied to MRI image stacks, and then expressed as a ratio to provide a measure of disease burden in each animal, as previously described [30, 31]. Analyses of lesion volume on MR images were performed with the investigators reading the images blind to treatment groups.

### 2.10 Pathology studies

#### 2.10.1 Gross examination following fixation

The fixed lungs were sliced serially and lesions counted as described previously^31^. Each lung lobe was evaluated separately and discrete lesions in the parenchyma were counted. Where lesions had coalesced, these were measured and recorded. Lung-associated lymph nodes, particularly around the tracheal bifurcation, were dissected and examined. The remaining tissues were examined during trimming.

#### 2.10.2 Histopathological examination

Representative samples from each lung lobe and other organs were processed to paraffin wax, sectioned at 3–5 μm, and stained with haematoxylin and eosin (HE). For each lung lobe, tissue slices containing obvious lesions were chosen for histological examination. Where gross lesions were not visible, a sample was taken from a pre-defined anatomical location from each lobe to establish consistency between animals. The nature and severity of the microscopic lesions was evaluated subjectively by a pathologist who was blinded to the treatment groups to prevent bias. Lesions were classified according to the scheme used by Lin [34]. In non-pulmonary tissue, the occurrence of tuberculosis induced lesions was scored as present or absent.

### 2.11 Bacteriology

The spleen, kidneys, liver and tracheobronchial lymph nodes were sampled for the presence of viable *M*. *tuberculosis* post-mortem^31^. Where available, tissue sections with and without visible tuberculous lesions were collected for analysis. Weighed tissue samples were homogenized in 2 ml of sterile water, and either serially diluted in sterile water prior to being plated or plated undiluted directly onto Middlebrook 7H11 OADC selective agar. Plates were incubated for three weeks at 37°C and resultant colonies were confirmed as *M*. *tuberculosis* and counted. Mean Colony forming unit (CFU) per gram from each tissue sample were determined.

### 2.12 Statistical analyses

Differences in the pathology scores, pulmonary disease measures and clinical measures of disease burden at the end of study were compared between test groups using the non-parametric Mann-Whitney U test, in Graphpad Prism, version 5.01 (GraphPad Software Inc, La Jolla, California, USA). The Spearman correlation test was used to determine the level of correlation between study parameters using GraphPad Prism, version 5.01 (GraphPad Software Inc, La Jolla, California, USA).

## 3. Results

### 3.1 *M*. *tuberculosis* exposure and disease progression post-challenge

**Fig 1:**
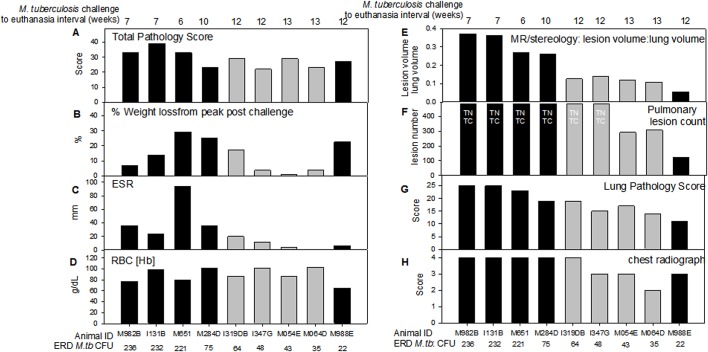
Measures of pulmonary disease and clinical disease severity in Mauritian cynomolgus macaques following aerosol exposure to a range of doses of *M*. *tuberculosis*. Black fill colour represents animals in which disease progressed rapidly and met endpoint criteria prior to the end of the study. Panel A: the total pathology score determined using a quantitative scoring system; Panel B: % weight loss from peak post-challenge weight on the day of euthanasia; Panel C: Erythrocyte sedimentation rate (ESR) on the day of euthanasia, or seven days prior to euthanasia (M982B, I131B, M988E); Panel D: Red blood cell haemoglobin concentration (RBC[Hb]) on the day of euthanasia; Panel E: the lung to lesion volume ratio determined using MR stereology; Panel F: number of lesions in the lung enumerated by serial sectioning and manual counting; Panel G: the scores attributed to the pulmonary component as part of the total pathology score; Panel H: score attributed to the chest radiogram on the day of euthanasia; TNTC indicates too numerous to count.

Nine Mauritian cynomolgus macaques were exposed to aerosols containing a planned wide spread of estimated retained doses of *M*. *tuberculosis* ranging from 22–236 CFU (Table 1) and monitored for changes in behaviour and clinical parameters for up to 13 weeks after challenge. Disease progressed in five of nine animals to a level that met the humane endpoint criteria for moderate disease. In the three macaques (M982B, I131B, M651) that received the very high estimated retained aerosol doses of *M*. *tuberculosis* (236, 232, and 221 CFU), disease progressed rapidly and they were euthanized six or seven weeks after challenge. In the days prior to euthanasia, all three animals showed changes in their behaviour, including reduction in feeding and drinking, increased respiratory rates, ruffled coat condition. Two of the three (M982B and I131B) also became withdrawn or depressed and coughing was observed (Table 2). All three macaques also showed abnormal thoracic radiographs and clinical parameters that deviated from normal ranges that included weight loss, anaemia, and increased ESR (Fig 1).

**Table 2:** Contra-indicators observed in cynomolgus macaques of Mauritian origin following aerosol infection with *M*. *tuberculosis*.

Animal I. D.	Estimated Retained Dose *M*. *tuberculosis*	Indicators observed post challenge. (week post challenge indicator recorded)		Indicators observed prior to euthanasia			
		Respiration rate	Cough	Behaviour	Eating / drinking	Respiration rate Under sedation	Other indicators
M982B	236	Rapid	Yes	Depressed withdrawn	No / No	Rapid / Shallow	Very ruffled coat. Thin
		(5–6)	(8)				
I131B	232	Rapid	Yes	Depressed	No / No	Rapid / Shallow	Very ruffled coat
		(5–6)	(5)				
M651	221	Rapid	No	Normal	No / No	Rapid / Laboured	Ruffled coat, Thin
		(5–6)					
M284D	75	Rapid	Yes	Normal	Normal	Rapid / Shallow	NAD
		(6–10)	(6, 7, 8)				
I319DB	64	Rapid	Yes	Normal	Normal	Rapid laboured	NAD
		(7–12)	(6, 7, 8)				
I347G	48	Rapid	Yes	Normal	Normal	Rapid / Shallow	NAD
		(6–12)	(6, 7, 8)				
M054E	43	Rapid	Yes	Normal	Normal	Normal	NAD
		(7–12)	(6, 7, 8)				
M064D	35	Rapid	Yes	Normal	Normal	Normal	NAD
		(7–12)	(6, 7, 8)				
M988E	22	Rapid	Yes	Depressed Withdrawn	Normal	Rapid	NAD
		(7–12)	(6, 7, 8)				

NAD: No abnormalities detected.

The macaques which received the six lower doses of *M*. *tuberculosis* developed increased respiratory rates between week 6 and 12 after aerosol exposure and cough during weeks seven, eight and nine. Ten weeks after challenge, macaque M284D, who received a high estimated retained dose (75 CFU) of *M*. *tuberculosis*, was euthanized as the level of disease had progressed to meet humane end point criteria. The five macaques that were exposed to medium or low doses of *M*. *tuberculosis* exhibited normal behaviour and changes in weight, haemoglobin level, temperature ESR that did not exceed the normal limits, although abnormalities were detected on thoracic radiographs (Fig 1). These five animals were euthanized as planned, during the 12^th^ (M988E, I319D, I347G) or 13^th^ (M054E, M064D) week after challenge. Macaque M988E, that was exposed to the lowest dose of *M*. *tuberculosis* (22 CFU), became withdrawn and on the day of euthanasia, exhibited chronic anaemia and a body weight loss in line with endpoint criteria.

### 3.2 IFN-γ response following aerosol challenge with *M*. *tuberculosis*

**Fig 2:**
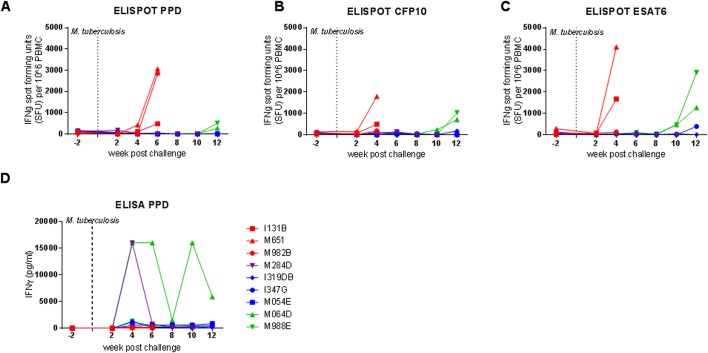
Immune responses after aerosol challenge with *M*. *tuberculosis*. The frequency of *Mycobacterium-*specific IFN-γ secreting cells measured after challenge by ELISpot is shown in panels A (PPD), B (CFP10) and C (ESAT6). Panel D shows the profile of IFN-γ secretion by PPD-stimulated whole blood measured by ELISA. Exposure to *M*. *tuberculosis* at week 0 is indicated by the dotted line.

The *Mycobacterium*-specific IFN-γ response was measured by ELISpot (Fig 2A, 2B and 2C) and ELISA (Fig 2D) applied at two weekly intervals throughout the study. High frequencies of IFN-γ producing cells measured by ELISpot were detected four to six weeks after challenge in the three macaques (M982B, I131B, M651) exposed to the highest doses of *M*. *tuberculosis*. The peak response to PPD was detected six weeks after challenge, while the highest responses to ESAT6 and CFP10 were measured four weeks after challenge; however, responses to these two antigens were not measured at week six in these three animals as cells were not available. Of the six macaques exposed to the lower doses, responses remained low until ten or twelve weeks after challenge, when M988E and M064D made responses above pre-infection levels to all three antigens, in contrast I319DB responded to ESAT6 and CFP10 but not PPD, while M284D responded to CFP10 and PPD. Responses to PPD, ESAT6 or CFP10 above pre-infection levels were not detected in I347G and M054E following exposure to *M*. *tuberculosis*. Evaluation by ELISA of blood collected from M064D at four, six and ten weeks and from M284D at four weeks after challenge, produced high levels of IFN-γ following stimulation with PPD, which was in contrast to the very low levels secreted by blood collected from the other seven animals during the study.

### 3.3 Measures of tuberculosis-induced disease burden

At the end of the study, the level of *M*. *tuberculosis*-induced disease burden was determined using a range of approaches that have been used previously for the evaluation of TB disease in both rhesus [30, 31] and cynomolgus macaques [31] (Fig 1). *M*. *tuberculosis* -induced disease burden measured by total pathology score (Fig 1A) and the clinical markers of weight loss (Fig 1B), increased in ESR (Fig 1C) and reduction in red blood cell haemoglobin concentration (Fig 1D) showed non-significant trends to be higher in the four animals exposed to very high, or high aerosol doses of *M*. *tuberculosis* relative to those receiving lower dose challenge. Furthermore, these high and very high dose animals developed symptoms indicative of progressive disease and met humane endpoint criteria, whilst the five animals exposed to lower doses controlled disease progression to the end of the study.

**Fig 3:**
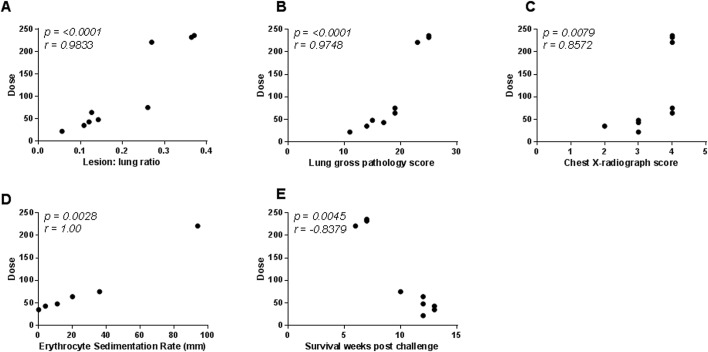
Correlation plots comparing the challenge dose (estimated retained) of *M*. *tuberculosis* with disease outcome measured by either lesion volume to lung volume ratio (A), lung gross pathology score (B), Chest radiograph score (C), erythrocyte sedimentation rate (D), or survival time post challenge (E). Data points represent individual animals, and vaccination groups are indicated by colour. Spearman’s correlation coefficient (*r*) and significance values (*p*) are indicated.

**Fig 4:**
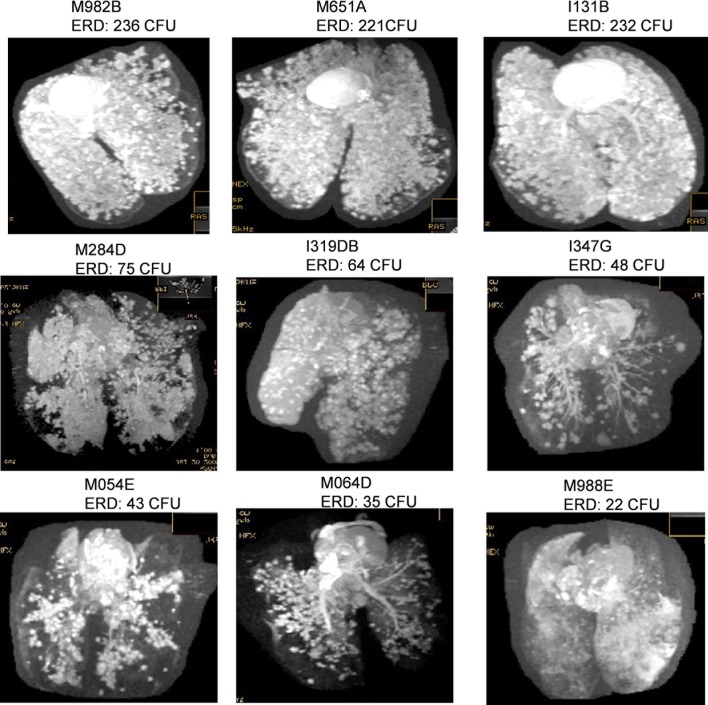
Magnetic resonance (MR) images of lungs from Mauritian cynomolgus macaques following aerosol infection with a range of doses of *M*. *tuberculosis*.

The level of pulmonary disease measured by MR stereology, gross pathology score and chest radiograph score was significantly higher in the four macaques that were exposed to very high or high challenge doses than in the macaques exposed to medium or low doses of *M*. *tuberculosis* (MR stereology: *p = 0*.*0159*, gross pathology score: *p = 0*.*0238*, chest radiograph score: p = 0.0479) and increased in line with the exposure dose (Fig 1E–1H). The Spearman’s Rank correlation test revealed significant relationships between exposure dose and lung disease burden quantified by MR stereology (*p = <0*.*0001*, *r = 0*.*9833*), lung gross pathology (*p = <0*.*0001*, *r = 0*.*9748*), chest radiograph score (*p = 0*.*0079*, *r = 0*.*8572*), ESR (*p = 0*.*0028*, *r = 1*.*00*) (Fig 3A, 3B, 3C and 3D) and survival time after challenge (Fig 2E). Three-dimensional images constructed from the MR scans demonstrated the relationship between disease burden and exposure dose and showed the lesions to be evenly distributed throughout all of the lobes of the lung (Fig 4). Macroscopic examination confirmed the pulmonary lesions in animals exposed to higher doses of *M*. *tuberculosis*, to be more severe than in animals receiving lower dose challenge (Table 3). An atypical combination of mature mild lesions with very numerous, small miliary lesions was noted in animal M988E.

**Table 3:** Summary of lung lesions at dissection of fixed lungs.

Animal #	Dose (cfu)	Discrete lesions		Coalesced lesions		Range of size of coalesced lesions (mm)	Comments
		right	left	right	left		
M982B	236	TNTC	250	TNTC	18	80% consolidation of right upper 1; 25 x 20 x 15 lesion in right middle. A whole section of left lobe occupied by coalesced lesions.	Multifocal granulomatous pneumonia with extensive coalesced lesions. Fibrous adhesions between lobes. Hilar lymph nodes enlarged and caseous.
I131B	232	TNTC	TNTC	TNTC	TNTC	80–90% consolidation of left lung	Multifocal granulomatous pneumonia with extensive coalesced lesions. Fibrous adhesions between lobes. Left lobes fused
M651A	221	TNTC	TNTC	TNTC	TNTC	80–90% consolidation of left lung	Multifocal granulomatous pneumonia with extensive coalesced lesions. Fibrous adhesions between lobes and with parietal pleura. Left lobes fused
M284D	75	TNTC	TNTC	TNTC	TNTC	8 X 6 to 22 x 12	Lesions too numerous to count and too extensive to measure in right and left lobes. Consolidation estimated as 70–80%. Severe pathology.
I319DB	64	224	TNTC	37	TNTC	4 x 6 to 15 x 18	Lesions too numerous to count and too extensive to measure in left lobes. Consolidation estimated as 80–90%. Widespread fibrous adhesions between upper and lower lobes of right and left lungs. Moderate/severe pathology
I 347G	48	TNTC	116	TNTC	9	5 x 4 to 30 x 30	Lesions too numerous to count and too extensive to measure in left lobes. Consolidation estimated as 86–80%. Fibrous adhesions between right lung lobes. Moderate/severe pathology.
M054E	43	150	110	17	15	5 x 5 to 12 x 15	Multifocal granulomatous pneumonia with coalesced lesions. Mild/moderate pathology.
M064D	35	174	110	11	13	5 x 5 to 12 x 10	Multifocal granulomatous pneumonia with coalesced lesions. Mild pathology
M988E	22	74	36	3	10	5 x 8 to 20 x 15	All lobes contained multiple miliary lesions that were too small and numerous to count. Fibrous adhesions were present between left upper and lower lobes. Atypical pathology

TNTC: Too numerous to count.

Sections of lung, hilar lymph node, liver, hepatic lymph node, kidney and spleen from all nine animals were microscopically examined for the presence of tuberculous lesions, and granulomas were classified according to the scheme used by Lin *et al* [34] (Table 4). Tuberculous granulomas varying in nature from unorganised to caseated were detected in all animals; caseated lesions were more extensive in animals that received the higher doses. Calcification of caseated tissue was observed in I319DB (lung), M064D (lung) and M988E (spleen). M988E was exceptional, in that superimposed on the caseated granulomas was what appeared to be a more recent development of numerous, small, multifocal granuloma, principally present in lung, liver and kidney.

**Table 4:** Summary of granuloma distribution.

Animal	Dose (cfu)	Tissue	Type of granuloma				
			Unorg	Solid	Neutro	Caseated	Coalesced
M982B	236	Hilar LN			**+**	**+**	
		Lung	**+**	**+**	**+**	**+**	
		Liver					
		Kidney			**+**		
		spleen			**+**	**+**	
I131B	232	Hilar LN				**+**	
		Lung				**+**	**+**
		Liver	**+**	**+**			
		Kidney		**+**		**+**	
		spleen	**+**	**+**	**+**		
M651A	221	Hilar LN				**+**	
		Lung	**+**	**+**	**+**	**+**	**+**
		Liver	**+**	**+**	**+**	**+**	
		Kidney	**+**	**+**	**+**	**+**	
		spleen	**+**	**+**	**+**	**+**	
M284D	75	Hilar LN				**+**	
		Lung	**+**	**+**	**+**	**+**	**+**
		Liver	**+**				
		Hepatic LN				**+**	
		Kidneys					
		Spleen					
I319DB	64	Hilar LN				**+**	
		Lung	**+**	**+**	**+**	**+**	**+**
		Liver	**+**	**+**	**+**		
		Kidneys				**+**	
		Spleen					
1347G	48	Hilar LN				**+**	
		Lung	**+**	**+**	**+**	**+**	
		Liver				**+**	
		Kidneys				**+**	
		Spleen				**+**	
M054E	43	Hilar LN				**+**	
		Lung	**+**	**+**	**+**	**+**	**+**
		Liver	**+**	**+**			
		Hepatic LN				**+**	
		Kidneys					
		Spleen	**+**	**+**	**+**	**+**	
M064D	35	Hilar LN				**+**	
		Lung	**+**	**+**	**+**	**+**	
		Liver	**+**				
		Hepatic LN	**+**			**+**	
		Kidneys					
		Spleen	**+**			**+**	
M988E	22	Hilar LN				**+**	
		Lung	**+**	**+**	**+**	**+**	**+**
		Liver	**+**	**+**			
		Kidneys	**+**		**+**	**+**	
		Spleen				**+**	

Unorg: Unorganised; Neutro: neutrophic.

### 3.4 Extra-pulmonary organ-specific bacterial burden

**Fig 5:**
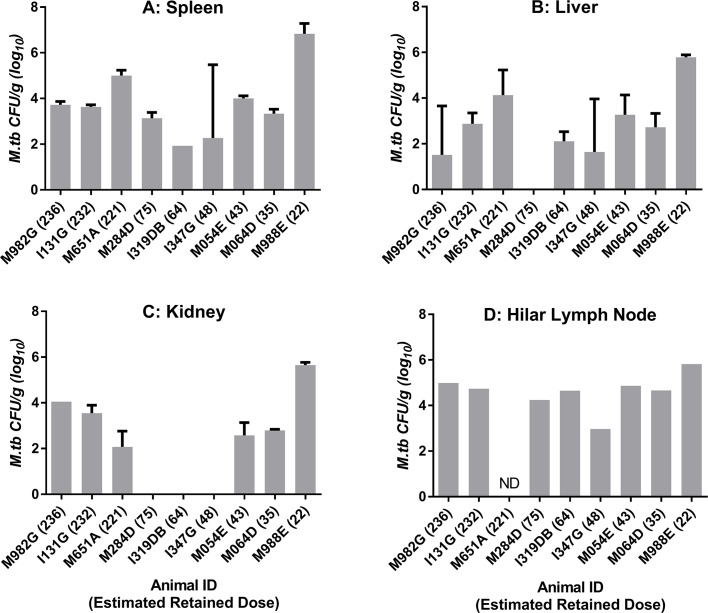
Bacterial burden in extra-pulmonary tissues collected from Mauritian cynomolgus macaques following aerosol infection with a range of doses of *M*. *tuberculosis*. Bars show CFU recovered from samples of spleen (A), liver (B), kidney (C) and Hilar lymph node (D). CFU recovered from replicate tissue samples are shown as a mean with standard deviation indicated as error bars. Samples for which bacteriology analysis was not done (ND) are indicated.

The level of bacterial burden was evaluated in the liver, spleen, kidneys and hilar lymph nodes in all animals. A similar frequency of isolation from tissues and level of bacterial burden was seen across all animals although there was a trend for tissues collected from M998E to have higher bacterial loads than comparable tissues from the other eight macaques (Fig 5).

## 4. Discussion

This is the first report of the effect of exposure dose on the outcome of infection with *M*. *tuberculosis* in a more genetically homogeneous species of non-human primate. Mauritian cynomolgus macaques were susceptible to aerosol exposure to *M*. *tuberculosis*, and displayed clinical signs and disease features similar to those reported in rhesus and Chinese cynomolgus macaques following exposure to the same *M*. *tuberculosis* strain [30, 31]. All of the challenged Mauritian cynomolgus macaques exhibited clinical signs typical of active human tuberculosis and developed lung lesions with both a gross and microscopic pathology similar to that described previously in TB-infected rhesus and Asian macaques [4, 5, 31, 34]. The key features of the pathological changes were consistent with those described in human infections including a heterogeneity in lesion type and distribution, within and between individuals [35].The pattern of pulmonary disease was typical of experimental aerosol exposure with lesions distributed throughout the lung [31, 36] while the burden of disease was directly related to the aerosol exposure dose.

In order to make a direct comparison of the disease induced in Mauritian cynomolgus macaques with that in Chinese cynomolgus and rhesus macaques, an analysis of previously published studies was made where the only variable was the macaque type. Whilst the numbers in this study are small, the findings suggest that Mauritian cynomolgus macaques have only a limited ability to control disease, which appears to be lower than that of Chinese cynomolgus macaques and equivalent to, or potentially even less than that of rhesus macaques (Table 5). The very high presented aerosol exposure doses (1544–1650 CFU) applied to macaques M982B, I131B, and M651 were selected to match the doses presented (1470–1575 CFU) to rhesus macaques and Chinese cynomolgus macaques in a previously reported study [31] to allow direct comparison of susceptibility among macaque types. Dose comparison between these studies was performed on the basis of the ‘presented dose’ of *M*. *tuberculosis* rather than the ‘estimated retained dose’, due to refinements only more recently applied to the method to estimate the retained dose [8]. Unlike the three Chinese cynomolgus macaques who controlled disease during the thirteen weeks after very high dose challenge, but similar to the Indian rhesus macaques, progressive disease developed rapidly and endpoint criteria were met seven weeks after challenge in all three of the Mauritian cynomolgus macaques. Both rhesus and Chinese cynomolgus macaques controlled the disease induced following exposure to a high dose challenge of 630 CFU, the lowest dose presented in the study reported by Sharpe et al [31], for the full 13 week post-exposure period. In contrast, Mauritian cynomolgus macaque M284D, the only animal in this study to receive an equivalent high challenge dose, developed disease that met end point criteria ten weeks after challenge and showed a higher level of pulmonary disease measured by lesion: lung volume ratio than that measured in either rhesus or Chinese cynomolgus macaques exposed to similar doses. It is noteworthy that the clinical signs of cough and altered respiratory rates occurred in all Mauritian cynomolgus macaques exposed to high, medium and low doses of TB, but only occurred in a proportion of Indian rhesus macaques and were not seen in Chinese cynomolgus macaques after high dose challenge. Furthermore, one of the Mauritian cynomolgus macaques, (M988E) that received a low challenge dose developed miliary TB which perhaps suggests a lack of ability to adequately control primary infection. Thus, the Mauritian cynomolgus macaques displayed a response to infection which was more like that of rhesus macaques which has been described as a response resembling active TB in susceptible humans [4]. While further work is needed to fully elucidate the difference between these macaque populations, it is clear that geographic origin, as well as species, are important considerations in study design.

**Table 5:** Clinical features following aerosol exposure to *M*. *tuberculosis* in three macaque populations.

Species	Number in group	Presented Dose range (CFU)	Frequency of contra-indicators % of group showing feature						Lung disease	Total disease burden
			Abnormal Behaviour	Abnormal respiration	In-appetence	Cough	Other indicators	Survival < 12 weeks	Mean Lesion volume: lung volume (range)	Mean Total gross pathology score (range)
MCM	3	Very high	67%	100	100	67	100	100	0.33	35
									(0.27–0.37)	(33–39)
CCM	3		0	33	0	67	0	0	0.15	29.3
		[>1400]							(0.04–0.31)	(22–39)
IRM	3		100	100	67	100	100	100	0.23	29.7
									(0.13–0.38)	(21–28)
MCM	1	High	0	100	0	100	0	100	0.28	23
CCM	9		0	0	0	0	0	0	0.04	16.3
									(0.01–0.14)	(2–29)
IRM	6	[500–1400]	17	17	17	34	50	17	0.04	19.5
									(0.02–0.8)	(14–23)
MCM	3	Medium [250–500]	0	100	0	100	0	0	0.13	26.7
									(0.12–0.14)	(22–29)
MCM	2	Low	50	100	0	100	50	50	0.08	25
		[<250]							(0.06–0.11)	(23–27)

MCM: Mauritian cynomolgus macaque; CCM: Chinese cynomolgus macaque; IRM: Indian rhesus macaque. Other indicators: coat condition, activity level. Data on IRM and CCM from Sharpe *et al* [31]

The Mauritian cynomolgus macaque has been of particular value in HIV vaccine research [37] where MHC homogeneity has reduced the variability between animals after vaccination and has enhanced comparisons of vaccine regimens. In addition, the improved consistency in outcome has facilitated the use of fewer animals to obtain statistically significant results than if cynomolgus macaques of Chinese or Vietnamese origin were used [37]. In this study, the Mauritian cynomolgus macaques showed less variation in pulmonary disease burden measured as the ratio of lesion volume to lung volume ratio and total gross pathology scores within exposure dose groups, than in either Indian rhesus macaques or Chinese cynomolgus macaques (Table 5). These preliminary observations support the hypothesis that Mauritian cynomolgus macaques could provide a model with a more homogeneous outcome of TB infection. More work is needed to define the variation in outcome of exposure to low or ultra-low aerosol doses and establish an optimal dose for challenge in vaccine efficacy studies.

The Mauritian cynomolgus macaque offers the potential to be an important additional model that could greatly assist the search for correlates of protection and biomarkers of disease that would have an enormous impact on the evaluation of new TB vaccines. Similar to rhesus macaques and Asian cynomolgus macaques, Mauritian cynomolgus macaques made mycobacteria-specific IFNγ responses following exposure to *M*. *tuberculosis* with the frequency of IFNγ-producing cells reflecting disease burden and therefore antigen load [8]. However it is the differences in the immune responses made by different macaque populations that provide an important opportunity that could assist with the elucidation of new biomarkers of disease and/or correlates of protection. Further work with state-of the-art immunological tools will be required to fully characterise the response of the Mauritian cynomolgus macaque to *M*. *tuberculosis* infection and allow cross genotype and species comparisons to expose the mechanisms responsible for the differences in TB control between macaque species and populations

The genetic homogeneity of the Mauritian cynomolgus macaque population is an asset that offers the opportunity to further understand the immune response to vaccination and infection. The relative ease of MHC haplotype characterisation and the high frequency of selected MHC haplotypes have allowed population studies to be conducted in the HIV field. Possession of specific MHC class I and II haplotypes have been shown to influence the outcome of experimental infection of Mauritian cynomolgus macaques with SHIVSF162P4cy [38], together with the immune response induced [39], and these can be associated with superior viraemic control of SIV or SHIV viruses in naïve-challenged and vaccinated individuals [25–27]. Although the small size of this study prevented meaningful evaluation of the possession of specific MHC haplotypes on outcome of *M*. *tuberculosis* infection, statistical power will increase as more studies using this genetic sub-type of the species, presenting the opportunity to identify potential associations.

In conclusion, this study has shown that Mauritian cynomolgus macaque are susceptible to aerosol challenge with *M*. *tuberculosis*, and develop patterns of disease similar to that reported in cynomolgus macaques of Asian origin but display an increased susceptibility and reduced ability to control disease progression. The predisposition to develop high burden disease, together with, the homogeneity of the occurrence of cough, make this type of macaque an attractive host for studies of TB transmission. The restricted genetic variability of the Mauritian cynomolgus macaque population provides an advantage in the battle to elucidate correlates of protective immunity. Further work is required to develop the Mauritian cynomolgus macaque as a model for the evaluation of TB vaccines, particularly the definition of low and ultra-low aerosol doses suitable for the assessment of vaccine efficacy. However, once established, the benefits of the Mauritian cynomolgus macaque population recognised by the HIV field could be unlocked for TB research.
